# Therapeutic Potential of Chlorhexidine-Loaded Calcium Hydroxide-Based Intracanal Medications in Endo-Periodontal Lesions: An Ex Vivo and In Vitro Study

**DOI:** 10.3390/antibiotics12091416

**Published:** 2023-09-07

**Authors:** Kadiatou Sy, Charlène Chevalier, Mickaël Maton, Ilham Mokbel, Séverine Mahieux, Isabelle Houcke, Christel Neut, Brigitte Grosgogeat, Etienne Deveaux, Kerstin Gritsch, Kevimy Agossa

**Affiliations:** 1 U1008, Controlled Drug Delivery Systems and Biomaterials, Inserm, CHU Lille, Université de Lille, 59000 Lille, France; mickael.maton@univ-lille.fr (M.M.); etienne.deveaux@univ-lille.fr (E.D.); kevimy.agossa@univ-lille.fr (K.A.); 2 Faculté d’Odontologie, Hospices Civils de Lyon, Pôle d′Odontologie, Université Lyon 1, Université de Lyon, 69372 Lyon Cedex 08, France; brigitte.grosgogeat@univ-lyon1.fr (B.G.); kerstin.gritsch@univ-lyon1.fr (K.G.); 3 UMR CNRS 5615 Laboratoire des Multimatériaux et Interfaces, Université Lyon 1, 69100 Villeurbanne, France; charlene.chevalier@univ-lyon1.fr (C.C.); ilham.mokbel@univ-lyon1.fr (I.M.); 4 U1286 Infinite, Institute for Translational Research in Inflammation, Inserm, CHU Lille, Université de Lille, 59000 Lille, France; severine.mahieux@univ-lille.fr (S.M.); isabelle.houcke@univ-lille.fr (I.H.); christelneut@nordnet.fr (C.N.)

**Keywords:** endo-periodontal lesions, intracanal medication, ion release, local drug delivery, periodontal cells

## Abstract

Endo-periodontal lesions are challenging clinical situations where both the supporting tissues and the root canal of the same tooth are infected. In the present study, chlorhexidine (CHX)-loaded calcium hydroxide (CH) pastes were used as intracanal medications (ICMs). They were prepared and tested on pathogens found in both the root canal and the periodontal pocket. Exposure to 0.5% and 1% CHX-loaded ICMs decreased the growth of *Porphyromonas gingivalis* and was effective in eradicating or inhibiting an *Enterococcus faecalis* biofilm. CH was injected into the root canal of extracted human teeth immersed in deionized water. CHX-loaded ICMs resulted in the transradicular diffusion of active components outside the tooth through the apex and the lateral dentinal tubules, as shown by the release of CHX (from 3.99 µg/mL to 51.28 µg/mL) and changes in pH (from 6.63 to 8.18) and calcium concentrations (from 2.42 ppm to 14.67 ppm) after 7 days. The 0.5% CHX-loaded ICM was non-toxic and reduced the release of IL-6 by periodontal cells stimulated by *P. gingivalis* lipopolysaccharides. Results indicate that the root canal may serve as a reservoir for periodontal drug delivery and that CHX-based ICMs can be an adjuvant for the control of infections and inflammation in endo-periodontal lesions.

## 1. Introduction

Endo-periodontal lesions (EPLs) are clinical situations where the periodontium (tissues surrounding the tooth) and the root canal (inner part of the tooth) of the same tooth are infected, inducing the destruction of the tooth attachment apparatus [1,2]. The prevalence of EPLs varies between 4.9% and 17.3% of patients and approximately 0.4% of teeth, depending on the criteria used [3,4,5]. EPLs are caused by polymicrobial infections that may originate in the periodontium or the pulpal tissues or both. These infections activate an inflammatory response that leads to local resorption of the alveolar bone supporting the tooth [6,7,8]. The mechanisms involved in tissue destruction include many cellular players (polymorphonuclear neutrophils [PMN], macrophages, TH1, TH2, and TH17 cells, B lymphocytes, and osteoclasts) and are regulated by molecular mediators such as IL-1β, IL-6, IL-17, TNF-α, and RANKL/OPG [9].

There are numerous anatomical or potentially pathological communication pathways between the pulp cavity (inside the tooth) and the external root surface. Anatomical pathways include the apical foramen, lateral/accessory canals, and dentinal tubules. In EPLs associated with trauma or iatrogenic factors, vertical root fractures, root resorption, or perforations may serve as potential pathways [7,8,10,11,12] and may contribute to the dissemination of pathogens and their by-products in the canal and the supporting tissues of the tooth. These may explain the similarities and interactions observed between the microbial populations of the periodontal pocket and the dental canal [1,5,13,14,15]. Indeed, up to 62.5% of the bacterial species are common to both sites [16]. Periodontal pathogens such as *Fusobacterium nucleatum*, *Tannerella forsythia*, and *Prevotella intermedia*, which are commonly found in active periodontal pockets, have also been detected in infected root canals [1,13,14,17,18]. *Porphyromonas gingivalis*, which is considered as the “keystone pathogen” in periodontitis due to its ability to orchestrate dysbiosis and hijack the host inflammatory response, has been identified in 30% of periodontal pockets and 10% of infected root canals [14], while *Enterococcus faecalis*, which is frequently associated with chronic endodontic infections and failed root canal treatments, is present in 20% of periodontal pockets and 10% of root canals in teeth affected by EPLs [14].

The treatment of EPLs is challenging and involves strict infection control in both endodontic and periodontal tissues [7,19,20,21]. Intracanal antimicrobial medications temporarily placed in the root canal are adjuvants to mechanical debridement of infected root canals [10,22]. Calcium hydroxide (CH) has long been considered the gold standard of intracanal medications. Its antimicrobial effect is related to its dissociation into calcium (Ca^2+^) and hydroxyl (OH^−^) ions in an aqueous medium, resulting in strong alkalinization of the medium, which alters bacterial membrane proteins [23]. Reported pH values vary between 12.5 and 12.8 [22]. Several studies have reported a significant reduction in the bacterial load of root canals filled with CH. Other biological effects attributed to CH include an anti-inflammatory and a pro-mineralizing potential [24,25]. Chlorhexidine (CHX) has also been proposed as an intracanal medication because of its strong antimicrobial properties and its affinity for dental tissues that allows for prolonged drug release [26,27]. Our group recently confirmed that a mixture of CH and CHX is effective against microorganisms such as *E. faecalis*, which is frequently considered as non-susceptible to CH alone [28].

The intracanal antimicrobial medications placement in a tooth affected by an EPL has yielded promising clinical results in terms of both endodontic and periodontal parameters. Raheja et al. found that the intracanal application of a 2% CHX gel for 14 days enhances the reduction of periodontal pockets [29]. Root canal obturations using a minocycline + mineral trioxide aggregate (MTA) significantly improve periodontal parameters after two years [30]. The periodontal healing benefit of ICMs may also be related to a potential anti-inflammatory effect. The application of a CH ICM for 30 days reduced LPS, MMP, IL-1 β, and TNF-α levels in periodontal pockets regardless of the bacteria load in the root canal and the periodontal pocket [31]. To explain these clinical effects, a few ex vivo studies have evaluated the physico-chemical changes that occur at the external root surface of teeth obturated with different ICMs. The pH increased at the root surface in most studies, suggesting that the OH^−^ ions produced by the dissociation of CH are able to diffuse across the root. However, the diffusion routes are not well understood. Altogether, these studies indicate that ICMs placed in the root canal may exert some biological effects outside the canal, resulting in positive effects on the periodontium. These findings may be highly relevant for the treatment of EPLs. However, the underlying mechanisms need to be elucidated [32,33,34,35].

The present ex vivo/in vitro study investigated the diffusion of ICMs outside the dental root to better understand the observed clinical effect on the periodontal environment. The antimicrobial effect of CHX-loaded CH pastes on bacteria in both the root canal and the periodontal pocket (*P. gingivalis* and *E. faecalis*) was evaluated, and the release of active components of the ICMs across the dental root was investigated. To further clarify the mechanisms that may explain the clinical effect of ICMs on periodontal healing, the biological responses of periodontal cells exposed to ICM extracts were also studied.

## 2. Results

### 2.1. Antimicrobial Properties of CH + CHX Intracanal Medications

#### 2.1.1. Bacterial Susceptibility to Chlorhexidine (Minimal Inhibitory Concentrations [MIC] and Minimal Bactericidal Concentrations [MBC]) and Time-Kill Assay

The MICs/MBCs of CHX for *E. faecalis* and *P. gingivalis* were determined, and the time–kill effect of CH + CHX mixtures on these bacteria was evaluated. The MICs/MBCs and time–kill curves for *E. faecalis* were reported in a previous publication by our group [28]. The MIC of *P. gingivalis* could not be determined because of the turbidity of the blood-supplemented medium. The low MBC (32 mg/L) confirmed that CHX has a bactericidal and bacteriostatic effect on *E. faecalis* and *P. gingivalis*.

**Figure 1:**
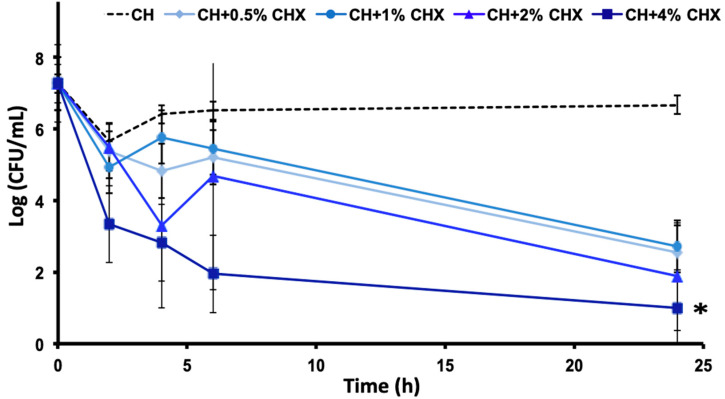
Time–kill kinetics curves and areas under the curves (AUC) of the time–kill kinetics for the CH + 0.5%, 1%, 2%, and 4% CHX solutions (*): significant difference (*p* < 0.05) between the CH formulation and the test formulations (ANOVA test).

The time–kill kinetics showed a dose-dependent reduction in the number of viable *P. gingivalis* cells (Figure 1) in a medium containing CH + CHX. CH + 4% CHX was the most effective medication compared to the control (CH alone) (*p* < 0.05).

#### 2.1.2. Antibiofilm Activity

The results of the tests on an *E. faecalis* biofilm are presented in Table 1. An inhibitory effect on biofilm formation (Table 1A) and an antibiofilm effect of CH on a mature biofilm (Table 1B) were observed at weeks 1, 2, 3, and 4, regardless of the CHX presence.

### 2.2. Diffusion of Active Components across the Dental Root

#### 2.2.1. Diffusion of Hydroxyl Ions

**Figure 2:**
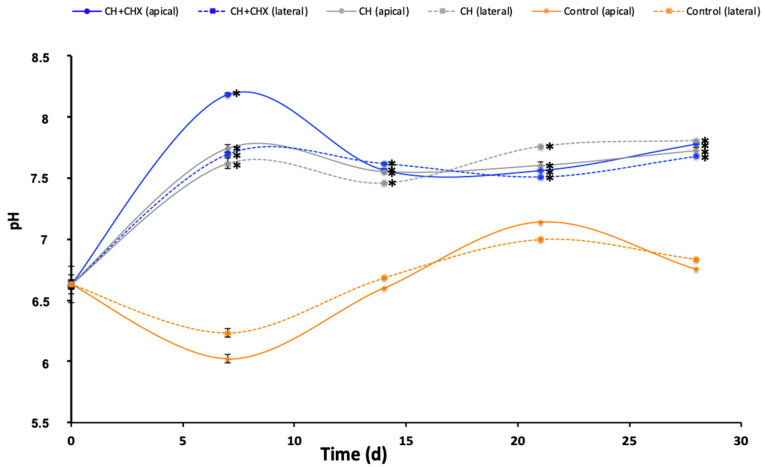
pH changes over 28 days in the diffusion medium of teeth not containing (control) or containing an intracanal medication (calcium hydroxide alone [CH] or CH + 1% chlorhexidine [CH + CHX]) through the apex (apical) or the dental tubuli (lateral). There was a significant difference (*) (*p* < 0.05) at all times between the control and the test formulations (CH and CH + CHX) (ANOVA test).

When the teeth containing a CH or a CH + CHX pastes were immersed in deionized water, the transradicular diffusion of OH^−^ ions resulted in a sharp increase in pH up to day 7 (Figure 2) followed by a plateau phase up to day 28. At day 7, the change in pH was more marked in the apical group than in the lateral group and with the CH + CHX paste than with the CH paste. At all times, the concentration of OH^−^ ions was significantly higher in the medium containing teeth with CH alone (pH 7.55 to 7.76) or CH + CHX (pH 7.51 to 8.18) compared to the control teeth (pH 6.23 to 7.14).

#### 2.2.2. Diffusion of Calcium Ions

**Figure 3:**
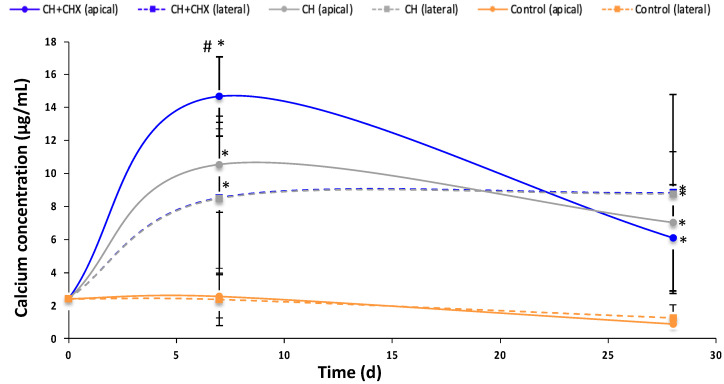
Changes in calcium ion concentrations in the diffusion medium of teeth not containing (control) or containing an intracanal medication: calcium hydroxide alone (CH) or CH + 1% chlorhexidine (CH + CHX), through the apex (apical) or the dental tubuli (lateral). There was a significant difference with the control group (*) (*p* < 0.05) and the CH group (#) (*p* < 0.05) (ANOVA test).

The release of Ca^2+^ ions was higher at day 7 in the apical group than in the lateral group (Figure 3). The addition of CHX tended to increase the release of Ca^2+^ ions at day 7 only. At days 7 and 28, more Ca^2+^ ions were released when CH− or CH + CHX-obturated teeth were immersed in the medium compared to non-obturated teeth (*p* = 4.66 × 10^−12^ at day 7; *p* = 3.90 × 10^−5^ at day 28).

#### 2.2.3. Diffusion of Chlorhexidine

**Figure 4:**
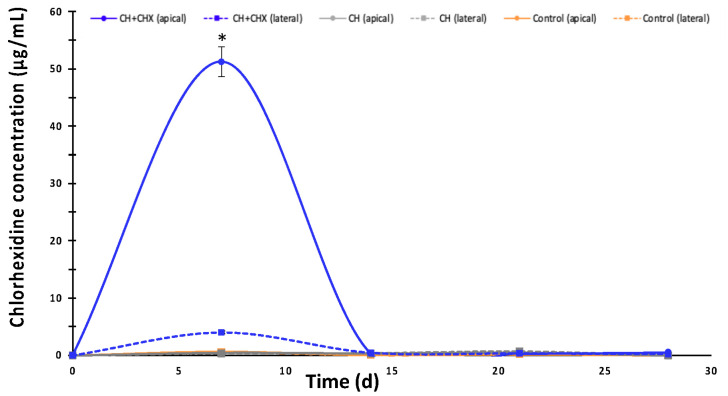
The evolution of chlorhexidine concentrations in the diffusion medium of teeth containing an intracanal medication: calcium hydroxide alone (CH) or CH + 1% chlorhexidine (CH + CHX) through the apex (apical) or the dentinal tubuli (lateral). Standard deviation: 95% confidence interval. (*): significant difference (*p* < 0.05) between the CH + CHX (apical) group and the other groups (ANOVA test).

On day 7 (Figure 4), a large amount of CHX was released through the apex of teeth filled with CH + CHX (51.28 μg/mL) and a much lower amount via the lateral route (3.99 μg/mL). No CHX release could be detected for all the other conditions and time points (<0.7 μg/mL (below the limits of quantification)).

### 2.3. Biological Effects

#### 2.3.1. Cell Viability

**Figure 5:**
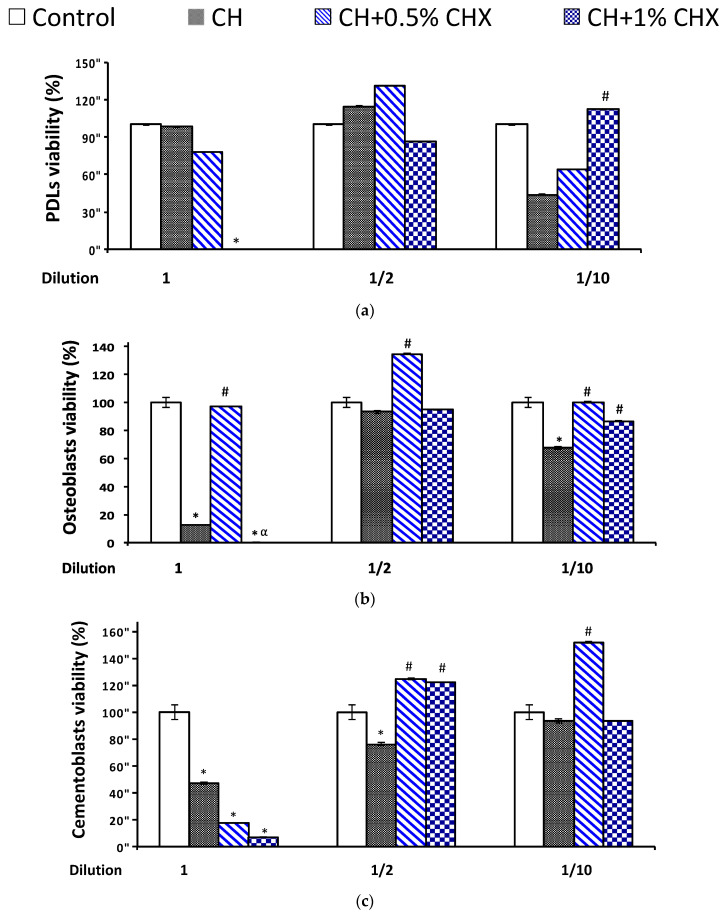
Proliferation of (**a**) periodontal ligament fibroblasts (PDLs), (**b**) osteoblasts, and (**c**) cementoblasts after a 1-day contact with 1, 1/2 or 1/10 diluted calcium hydroxide extracts with (CH + 0.5% CHX and CH + 1% CHX) or without chlorhexidine (CH) (*n* = 3). The measurements were determined using an Alamar Blue^®^ assay, and the data are expressed as means and standard deviations. Difference between metabolically inactive and metabolically active cells. Standard deviation: 95% confidence interval; (*) significant difference with control group (*p* < 0.05); (#) significant difference with CH (*p* < 0.05), (α) significant difference with 0.5% CHX group (*p* < 0.05) (ANOVA test).

The cell viability (close to or more than 80%) of PDLs, osteoblasts, and cementoblasts was acceptable with ½-diluted extracts regardless of the presence of CHX (124% to 134% with CH + 0.5% CHX and 76% to 114% without CHX [CH]) (Figure 5).

#### 2.3.2. Alkaline Phosphatase (ALP) Activity

**Figure 6:**
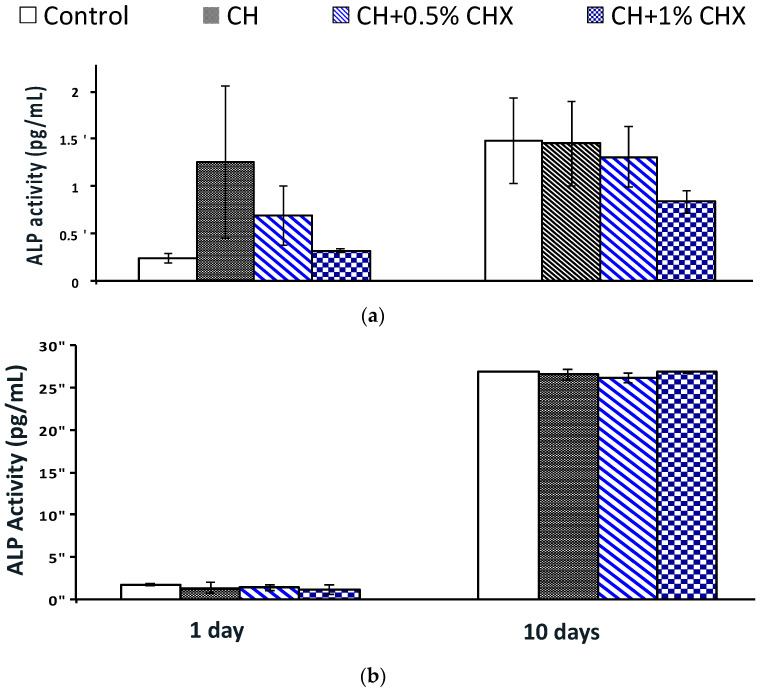
Intracellular alkaline phosphatase (ALP) activity of (**a**) osteoblasts and (**b**) cementoblasts stimulated with calcium hydroxide extracts with 0.5% chlorhexidine (CH + 0.5% CHX), or 1% chlorhexidine (CH + 1% CHX), or without chlorhexidine (CH) for 1 and 10 days (*n* = 3); no statistical difference (ANOVA test).

No significant intergroup differences were observed on days 1 and 10 with respect to ALP activity, which indicates that the CH or CH + CHX extracts have no effect on the early mineralization potential of osteoblasts (Figure 6a). A similar conclusion can be drawn for cementoblasts even though ALP activity increased significantly in all groups between days 1 and 10 (Figure 6b).

#### 2.3.3. Mineralized-Bone-Like Nodule Formation

**Figure 7:**
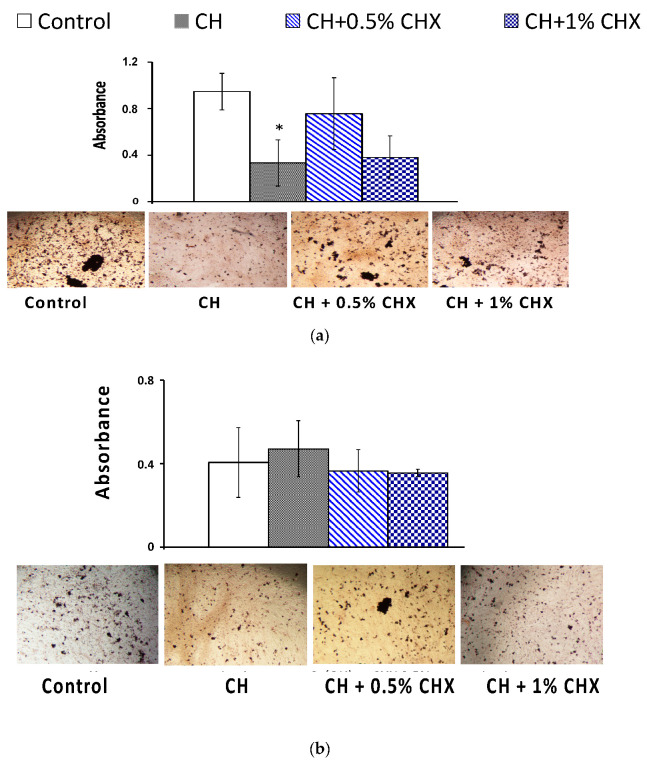
Semi-quantification of calcium deposits in (**a**) osteoblasts and (**b**) cementoblasts using Alizarin Red Staining after stimulation with calcium hydroxide alone (CH) or calcium hydroxide + chlorhexidine (CH + 0.5% CHX and CH + 1% CHX) for 14 days (*n* = 3) and corresponding images obtained using an optical microscope. Significant difference with control group (*) (*p* < 0.05) (ANOVA test).

The CH + CHX extracts (CH + 0.5% CHX and CH + 1% CHX) had no effect on the late mineralization of osteoblasts (Figure 7a). A significant reduction in the number of bone-like nodules was observed in the CH group compared to the controls (0.33 pg/mL and 0.95 pg/mL, respectively, *p* = 0.024).

The CH and CH + CHX extracts (CH + 0.5% CHX and CH + 1% CHX) had no effect on the late mineralization of cementoblasts (Figure 7b). The visual analysis of optical microscopic images and the results of the semi-quantitative method were consistent.

#### 2.3.4. Anti-Inflammatory Activity: TNF-α and IL-6 Levels

**Figure 8:**
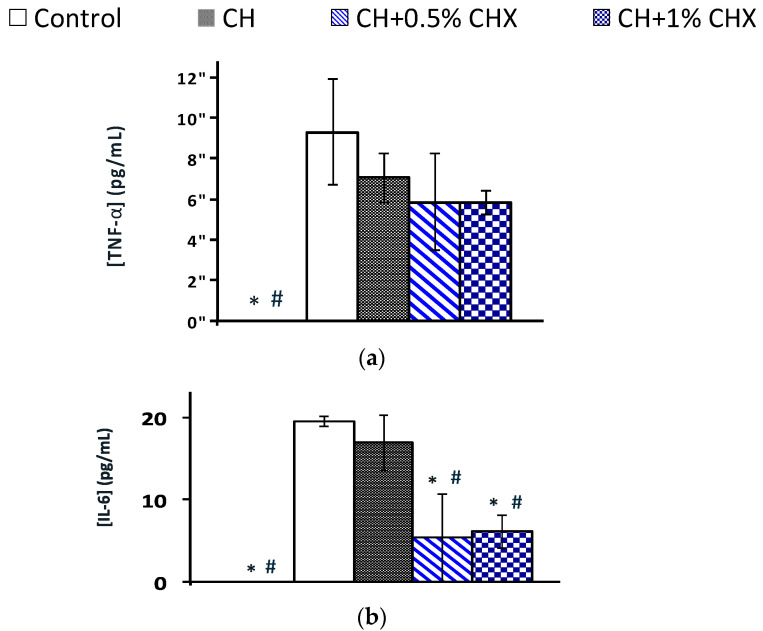
Expression of (**a**) tumor necrosis factor-alpha (TNF-α) and (**b**) interleukin 6 (IL-6) by periodontal ligament fibroblasts after a 24 h exposure to LPS followed by treatments with calcium hydroxide extracts with 0.5% chlorhexidine (CH + 0.5% CHX) or 1% chlorhexidine (CH + 1% CHX) or without chlorhexidine (CH), (*n* = 3); control+: culture control or positive control (cells not stimulated by LPS). Significant difference with control group (*) (*p* < 0.05); significant difference with CH (#) (*p* < 0.05) (ANOVA test).

CH + CHX extracts reduced TNF-α (Figure 8a) and IL-6 (Figure 8b) levels in the culture medium of PDLs stimulated with *P. gingivalis* LPS compared to CH alone or the control. The difference was significant for IL-6 (19.84 pg/mL, 16.89 pg/mL, and 5.33 to 6.08 pg/mL, respectively, for the control, CH alone, and CH + CHX). (*p* = 0.007) (Figure 8b).

## 3. Discussion

The primary aim of the present study was to investigate the effect of antimicrobial-containing, CH-based medications outside the dental root when the medications were initially placed in the root canal in order to explain the empirically observed clinical effect of ICMs on periodontal healing of EPLs [29,30]. Our results indicated that the active compounds in the ICMs can diffuse through the dental root via the apex (apical route) and the dentinal tubuli (lateral route). Importantly, not only Ca^2+^ and OH^−^ ions produced by the dissociation of the CH pastes were detected but the CHX mixed with the ICMs could also be quantified, which to our knowledge, has never been reported before. Interesting results were also obtained with respect to the kinetics and diffusion routes of the active compounds through the dental root wall. The amounts of active compounds released through the lateral route were more than 10 times lower than that of the apical foramen. This finding is consistent with the diameter of these communication routes [36,37]. In terms of release performance, our team calculated that for 1 g of CH + CHX paste placed in the root canal, 0.119 mg of CHX is released apically and 0.014 mg laterally, which correspond to 1.19% and 0.14% of the initial drug load. Some studies have suggested that CHX is completely degraded due to the alkaline pH of CH when these two products are mixed together [38,39,40]. Our results do not support this hypothesis as CHX was detected after 7 days in the diffusion medium of teeth obturated with CH + CHX. It can be assumed that the buffering capacity of dentin may have limited or slowed CHX degradation by limiting the alkalinization of the medium. Furthermore, a recent review suggests that para-chloroaniline (pCA) formation, a by-product of the degradation of CHX, is more related to the high concentration of CHX than to chemical interactions with other molecules [41]. However, it is worth mentioning that measuring CHX levels was not possible in all the samples (3/10 apical and 3/10 lateral). The cause of this heterogeneity remains to be elucidated. We assume that age-related changes in dental structure may play a significant role in this aspect. Progressive narrowing of root canals and dentinal tubules due to the apposition of dentin and the occurrence of calcifications has been well documented in the literature [42,43], and this factor could not be controlled in our study.

Overall, the transradicular diffusion rate was higher in the earlier stages (up to day 7), and adding CHX increased the diffusion of Ca^2+^ and OH^−^ ions. Other studies have reported a similar quick, massive ion release induced by CH at day 7 (the so-called “burst” effect) [32,33,44,45,46,47], but, to our knowledge, no study has compared apical and lateral diffusion patterns. CHX appears to play an important role in controlling the release kinetics of CH-based ICMs. Carvalho et al. and Tanomaru et al. reported that ICMs prepared with a CHX solution release more HO^−^ and Ca^2+^ ions [32,48]. They attribute this effect to faster ionic dissociation when CHX is used in the aqueous form [32,49,50]. Conversely, when CHX is used in the gel form, the peak release of active compounds occurs later (day 30) [51,52]. This delay is thought to be related to the formation of calcium digluconate and a poorly soluble CHX precipitate [47,52,53]. In the control group, the immersion of dental roots without ICM resulted in slight pH variations that are probably attributable to the buffering effect of dentin [47].

In practice, temporary ICMs are placed in infected root canals to potentiate the effect of mechanical preparations by disinfecting areas not accessible to the instruments [10,22]. In this study, a dose-dependent potentiation of the antimicrobial action of CH on *P. gingivalis* is observed, which is found in both the root canal and the periodontal pocket and is not susceptible to CH alone. In a previous publication, our group reported similar results with *E. faecalis* [28]. The dosage used in the bacterial culture medium in contact with the ICMs confirmed that CHX is released at a concentration of 2.25 mg/L after 24 h, which is close to the MICs of these bacteria [28]. CH and two combinations of CH + CHX were both effective in inhibiting biofilm formation and eradicating mature biofilms. Like the studies by Delgado et al. and Balto et al., the *E. faecalis* biofilm in the present study was destroyed in the presence of CH [54,55]. These promising results may be explained by the high concentration of CH in contact with *E. faecalis*. The antimicrobial effect of CH is dose-dependent [56]. CH-based ICMs may complement the disinfection of areas inaccessible to instruments and slow down the recolonization of the canal during the inter-session period. It could be interesting for the clinician to have a specific ICM placed between appointments, that acts on the endodontic and periodontal tissues and contributes to EPL treatment.

Clinical and animal studies support the positive effect of CH on the healing of apical endodontic lesions [24,25]. However, whether this effect is attributable to the antimicrobial or to the non-antimicrobial properties of the CH is not well understood. The viability tests suggested that periodontal cells tolerate exposure to 1/2 dilutions of CH aqueous extracts containing 0.5 and 1% CHX, which indicates that low doses of CHX have a negligible effect on the toxicity of CH-based ICMs. The extracts showed no effect on the mineralization potential of osteoblasts and cementoblasts. Da Silva et al. similarly reported that CH and CH + 0.4% CHX has no effect on ALP activity or bone nodule number formation in rat calvaria osteoblast cultures [57], which may be related to the cell model used. In addition, CH extracts tend to reduce the production of TNF-α and IL-6 by periodontal ligament fibroblasts previously stimulated by *P. gingivalis* LPS (−24% for TNF-α and −13% for IL-6). This reduction was more important in the presence of CHX (−37% for TNF-α and −70% for IL-6) and was significant for IL-6. This effect could be explained by the cytokine degradation by alkaline hydrolysis of the amide bonds, as suggested in a previous study [58]. This is consistent with the accelerated CH dissociation in the presence of CHX resulting in the OH^−^ ions production and an increase in pH.

This experimental study presents some obvious limitations. The use of cell cultures and an ex vivo model are some of them. Other systems have been proposed that combine a diffusion model through the root with cell cultures [59]. Other limitations of the present study are related to the in vitro models used. First, the use of monospecies biofilms allows for easier implementation and good reproducibility [60]. However, the observations and results obtained with such models do not take into account the polymicrobial nature and complexity of the metabolic interactions found in biofilms associated with endodontic and periodontal pathologies [61]. Second, the microbial culture techniques used provide essential information but are limited to the number of live bacteria adherents on the experimental substrate or growing within the biofilm structure [62]. Although the use of multi-species biofilm is clearly of interest, monospecies models are still widely used in the endodontic literature. A literature review including 77 recent studies showed that in 86% of studies, a monospecies biofilm was cultured and *Enterococcus faecalis* was the most frequently used test species (in 79% of all studies, 92% of the monospecies studies) [63]. In addition, bacterial culture was the most frequently used quantification method (in 87% of the studies) [63]. Similarly, bacterial culture techniques including a time–kill test remain frequently used in endodontics to assess the effect of antimicrobial formulations or technologies [64,65,66,67,68,69].

Evidence of the diffusion of active compounds from the root canal to the root surface offers the possibility to extend the indications of intracanal medications to the treatment of endo-periodontal conditions. To this end, numerous areas are open to research, including: (i) the development of innovative scaffolds for controlled drug delivery. The use of hydrogels that are increasingly sophisticated but still easy to apply in the canal appears promising in this respect [70]. (ii) The development of novel experimental models is needed to investigate the effect of active ingredients on bacteria–cell interaction. For example, Pintor et al. recently developed a simplified model of primary root teeth that can be combined with a cell culture set-up. In this way, cells are stimulated under conditions closer to in vivo [71]. Animal models are also required to gain a better insight into the biological effects of intra-canal medications on the periodontium in order to optimize their performance. (iii) The research into alternative molecules to conventional antimicrobials is also a promising direction for the development of new generation intracanal medications [72]. Indeed, the complex pathophysiology of endodontic and periodontal conditions provides several potential molecular targets to modulate inflammation and tissue destruction. For example, a recent study highlights the positive effects of amelogenins on apical healing and pulpal regeneration when used as intracanal medication [73]. Further studies should be carried out using a more elaborate combined ex vivo/in vitro model to gain a better understanding of the pathophysiology of EPLs and the response of periodontal tissues in the presence of ICMs under conditions closer to reality.

## 4. Materials and Methods

### 4.1. Intracanal Medications Formulations

Two different formulations were used: (i) a formulation for testing the antimicrobial effect that was inserted in the canal to study diffusion and (ii) a formulation for preparing the extracts for the tests on the cells.

#### 4.1.1. Calcium Hydroxide Paste Formulation

CH paste was prepared by mixing CH powder (DentaFlux, Madrid, Spain) with distilled water (1:1 *w*/*v*) and spatulating the mixture on a glass plate. Antimicrobial-free CH paste was used as an ICM negative control. The test formulations were produced by replacing the distilled water with antimicrobial solutions of 0.5% or 1% CHX. The antimicrobial solutions were prepared by diluting 20% chlorhexidine digluconate (Evonik, Hanau, Germany) in sterile distilled water.

#### 4.1.2. Intracanal Medication Extract Preparation

Aqueous extracts were obtained by placing the CH alone, CH + 0.5% CHX, and CH + 0.1% CHX pastes (1 mL) at the bottom of 15 mL tubes. The pastes were then covered with 9 mL of serum-free Dulbecco’s Modified Eagle Medium (DMEM, Sigma-Aldrich, Saint-Quentin Fallavier, France) or Fibroblast Medium (FM, ScienCell^TM^, Carlsbad, CA, USA), depending on the cell type studied. They were incubated at 37 °C for 24 h according to International Organization for Standardization (ISO) 10993-Part 12 and ISO 10993-Part 18. The extracts were filtered through 0.45-μm sterile polyethersulfone filters (Nalgene^®^, Rochester, NY, USA) and were serially diluted with DMEM supplemented with 10% fetal bovine serum (FBS, Gibco^TM^, Thermo Fisher, Fisher Scientific, Waltham, MA, USA) or with FM supplemented with 10% FBS to give three concentrations (1/1, 1/2, 1/10 *v*/*v*) to determine the dose–response relationship.

### 4.2. Antimicrobial Properties of ICMs

The tests were performed using *E. faecalis* (C159-6), a facultative anaerobic Gram positive bacterium, and *P. gingivalis* (W83), an anaerobic Gram negative periodontopathogenic bacterium. These strains were isolated from clinical samples collected as part of a biological collection (IMPERIO, n° 2017-A02123-50) and were stored in the clinical bacteriology laboratory of the Faculty of Pharmacy, Université de Lille, France.

#### 4.2.1. Minimal Inhibitory Concentrations and Minimal Bactericidal Concentrations

The minimal inhibitory concentrations (MICs) and minimal bactericidal concentrations (MBCs) were determined using Clinical and Laboratory Standards Institute protocols (CLSI M07-A9, CLSI M26-A [74]). The broth microdilution method was used with 10 concentrations of the antimicrobial chlorhexidine digluconate solution and 24-well flat bottom microplates with an assay volume of 2 mL/well [75] (Supplementary Information S1).

#### 4.2.2. Time–Kill Kinetics Assay

A time–kill kinetics assay was performed to determine the time-dependent reduction in *P. gingivalis* CFUs caused by the antimicrobials, as previously reported [68,69,76]. Bacterial suspensions were exposed to the antimicrobials for different periods of time, and the CFUs of the surviving microbial populations were counted. Each formulation (1 mL) was placed in a 15 mL tube. Fresh brain–heart infusion broth (BH) (Oxoid, Basingstoke, UK) supplemented with defibrinated horse blood (E&O Laboratory, Burnhouse, UK) was added (8 mL) followed by 1 mL of *P. gingivalis* suspension (10^4^–10^6^ bacteria/mL). The tubes were then incubated under strict anaerobic conditions at 37 °C. At 0, 2, 4, 6, and 24 h, 100-μL samples were removed and were diluted 10-fold with cysteinated Ringer’s solution (Merck^®^, Darmstadt, Germany). The dilutions (100 μL) were seeded on Columbia Cysteine agar (CC) (Oxoid^®^) supplemented with defibrinated horse blood. The plates were incubated under strict anaerobic conditions for 24 h at 37 °C. The numbers of colonies were counted, and the results are expressed as log CFU/mL. All experiments were performed in triplicate, and the results are expressed as means ± standard deviations.

#### 4.2.3. Biofilm Tests

##### Antimicrobial Effect on Biofilm Formation and on Mature Biofilms

The antimicrobial effect of CH on *E. faecalis* biofilm formation and on *E. faecalis* mature biofilms were, respectively, investigated weekly for four weeks and by adding the CH formulations after three weeks of bacterial biofilm growth [77,78], adapted from a previously published protocol [79].

To investigate the antimicrobial effect of CH on *E. faecalis* biofilm formation, 300 µL of each formulation was deposited in the wells of four 24-well flat-bottom microplates (one for each week) and MH medium was added (1400 µL/well). The wells were then inoculated with 300 µL of bacterial (*E. faecalis*) solution per well, at a concentration of 10^6^ CFU/mL. The microplates were incubated at 37 °C under strict anaerobic conditions for one, two, three, or four weeks of culture. For the negative controls corresponding to wells without bacteria, the CH formulation was placed at the bottom of the well and the medium was added (1700 µL/well). Positive controls corresponded to wells with bacteria (300 µL/well) and medium (1700 µL/well). The medium was changed every 3–4 days [77]. Each time, the medium was removed, and the biofilms formed on the formulations were gently washed [67,68,79].

To study the antimicrobial effect of CH on *E. faecalis* mature biofilms, the formulations (CH or CH + CHX) were added after three weeks of bacterial biofilm growth [64,65]. First, MH medium was added (1700 µL/well) to 24-well flat-bottom microplates with a total volume of 2 mL per well. The wells were inoculated with 300 µL of bacterial culture (*E. faecalis*) per well, at a concentration of 10^6^ CFU/mL. The microplates were incubated at 37 °C under strict anaerobic conditions for three weeks to allow bacterial attachment. Negative controls (wells without bacteria) contained only medium (2000 µL/well). Positive controls contained 300 µL/well of bacteria and 1700 µL/well of medium. The medium was changed every 3–4 days [77]. Then, after biofilm formation (three weeks), the medium in each well was removed and the biofilms on the bottom were gently washed. The antimicrobial agent (CH or CH + CHX) (300 µL) was added to the corresponding wells with MH medium (1700 µL) and the microplates were incubated at 37 °C under strict anaerobic conditions for a further week.

##### Biofilm Quantification

For both experiments, after one, two, three, or four weeks of culture, the content of each well was mixed and 300 µL was withdrawn with a micropipette. An amount of 100 µL of the contents was placed on a Petri dish containing Mueller–Hinton agar (MHA) (Oxoid^®^), and 200 µL of the same sample was pipetted into BH broth for culturing. The broth and Petri dish were then incubated at 37 °C under strict anaerobic conditions. After 24–48 h, the colonies on the MHA plates were counted to assess viability. The turbidity of the BH broth cultures during the incubation period indicated bacterial growth.

### 4.3. Transradicular Release

#### 4.3.1. Tooth Preparation

A trans-radicular diffusion model was developed according to previous studies, [32,53,80,81] (Supplementary Information S2). Briefly, 60 previously extracted human permanent maxillary and mandibular anterior single-rooted teeth were used. No specific ethical approval was required according to French legislation (Public Health Code—Article L1245-2). Patients were informed of the possible re-use of the extracted teeth for research purposes and were given the opportunity to object.

**Figure 9:**
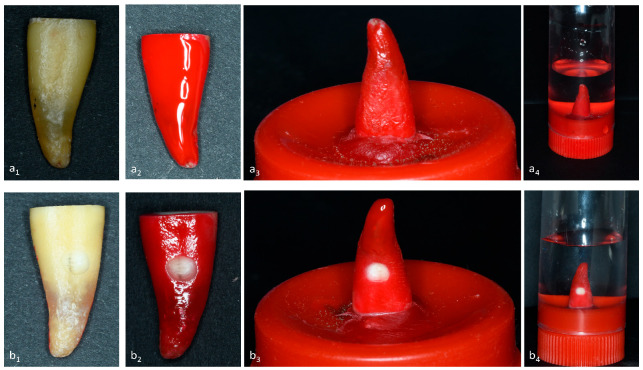
Diffusion model protocol with an incisor (IC) following chemo-mechanical preparation: (**a1**,**b1**) labio-lingual views, (**a1**) for apical diffusion and (**b1**) for lateral diffusion following the cavity preparation, (**a2**,**b2**), following the varnish application, (**a3**,**b3**) root attachment to the vial cap, and (**a4**,**b4**) immersion in 10 mL of deionized water and incubation at 37 °C in a humidified atmosphere.

The roots were randomly divided into two groups (Figure 9) of equal size (30 roots in each group). The first group (apical group) was used to assess apical diffusion though the apex. A gutta cone was placed in each canal and was extended slightly apically. Varnish layers were applied in the presence of the cone to avoid isolating the apical area [82]. The second group (lateral group) was used to assess lateral diffusion through the dental tubuli. A lateral cavity 1 mm in depth and 3 mm in diameter was made in the middle third of the root using a depth-marking cylindrical burr (Komet, Paris, France) (Figure 9) [80,82,83]. To remove the smear layer, the root surface cavities were filled with 3 mL of 17% EDTA for 1 min and were then rinsed with 3 mL of distilled water. The external surfaces of the root were covered with three layers of nail varnish, except for the prepared cavities.

Each group (apical and lateral) was then divided into three subgroups (*n* = 10): a subgroup filled with CH, a subgroup filled with CH + 1% CHX (CHX), and a subgroup without ICM as a negative control. The CH was placed in the canals using a counterclockwise motion of a #30 K-file and was then condensed with paper points to 12 mm from the root apex. Control teeth were filled with normal saline solution. Lastly, the coronal access was sealed with glass ionomer (Dentsply Maillefer Ballaigues, Switzerland). The coronal part of the roots was attached to the internal surface of a vial lid using sticky wax to avoid handling the roots during pH measurements. During test intervals, the roots were immersed in a vial containing deionized water and were stored at 37 °C for 28 days. The water was replaced every 7 days.

#### 4.3.2. Hydroxide Ion Release Analysis

pH measurements were performed using an HI9125 pH meter (Hanna Instruments, Lingolsheim, France) with an accuracy of 0.01. Hydroxyl ion quantification was performed by measuring the pH of 10-mL solutions at different times: days 0, 7, 14, 21, and 28 [81,83]. Three measurements were taken for each sample [82], and a calibration was performed every six measurements.

#### 4.3.3. Calcium Ion Release Analysis

The amount of Ca^2+^ ions released in the solutions was measured via inductively coupled plasma atomic emission spectroscopy (Vista-MPX CCD Simultaneous ICP-OES, Varian Inc., Palo Alto, CA, USA), as previously reported [32] (Supplementary Information S3). A calibration was performed between each group of measurements. Three replicates were collected for each measurement [84].

#### 4.3.4. Chlorhexidine Release Analysis

The amount of CHX released was determined via high performance liquid chromatography coupled with diode array detection (HPLC-DAD) (Shimadzu LC2040 I series, Kyoto, Japan) on days 0, 7, 14, 21, and 28 as previously described [85,86,87] (Supplementary Information S4). All the experiments were performed in triplicate, and the results are expressed as means ± standard deviations.

### 4.4. Biological Assessment

#### 4.4.1. Cell Isolation and Primary Cell Cultures

The cells tested were (i) human periodontal ligament fibroblasts (PDLs) derived from a human primary cell culture (#2630, ScienCell, USA); (ii) osteoblasts (MG63), an immortalized human cell line (MG63, CRL1427, ATCC) obtained from the American Type Culture Collection (ATCC^®^ CRL-1427™); and (iii) cementoblasts (OCM) derived from an immortalized mouse cell line (OCCM.30 mouse cementoblasts, Applied Biological Materials ([ABM Inc.], Richmond, BC, Canada).

The MG63 and OCM cells were cultured in DMEM and the PDL cells were cultured in FM. Rinses were performed with DPBS (Gibco^TM^, Thermo Fisher, Strasbourg, France). Control cells (culture and positive controls) were grown in DMEM or FM medium supplemented with 10% FBS, 5% penicillin/streptomycin, and 0.2% amphotericin B. For the FM medium, 1% growth factors were also added. All the cells were grown at 37 °C in a controlled atmosphere containing 5% CO_2_.

#### 4.4.2. Periodontal Cell Stimulation with Calcium Hydroxide Paste Extracts Containing Chlorhexidine

The day before each assay, each cell population was seeded in 200 μL of medium in 96-well microplates at a density of 10^2^ cells/mL or 1.25 × 10^3^ cells/mL, depending on the assay. The microplates were then incubated for 24 h at 37 °C in a controlled atmosphere containing 5% CO_2_.

On the day of the experiments, the media was removed from the microplates and was replaced with 200 μL of each extract [88,89]. The microplates were then incubated at 37 °C in a controlled atmosphere containing 5% CO_2_. The incubation time ranged from 1 to 14 days, depending on the assay. The medium was replenished with the extracts every 3–4 days. The control group consisted of wells containing cells alone. For each condition, one well contained only the extracts. The tests were performed in triplicate.

#### 4.4.3. Cell Viability

After 1 day of cells contact (10^3^ cells/mL) with the extracts, the contents of the wells were transferred to new 96-well microplates and 20 μL of Alamar Blue^®^ (DAL1025, Thermo Fisher Scientific) cell viability reagent was added directly to the wells at a final concentration of 10% (*v*/*v*). The microplates were then incubated at 37 °C for 5 h. The amount of resorufin formed was determined by measuring the absorbance at 570 nm with a 600 nm reference using a spectrometer (Infinite^®^ M200 PRO NanoQuant, Tecan, France). It was proportional to the number of live cells.

#### 4.4.4. Alkaline Phosphatase Activity

The osteoinduction potential was assessed via an alkaline phosphatase (ALP) activity colorimetric assay. ALP activity is an early marker of mineralization. Osteoblasts and cementoblasts were seeded in 200 μL of medium in 96-well microplates at 10^2^ cells/mL. After 1 and 10 days of culture, the extracellular amount of ALP was estimated in the cells supernatant using an ALP assay kit according to the manufacturer’s instructions (K412-500, BioVision Incorporated, Waltham, MA, USA). The assay has been described previously [57,90] (Supplementary Information S5).

#### 4.4.5. Mineralized Bone-like Nodule Formation

The remineralization activities of cementoblasts and osteoblasts (10^2^ cells/mL) were determined using Alizarin Red S (ARS) after 14 days, as previously described [91,92] (Supplementary Information S6).

#### 4.4.6. Anti-Inflammatory Activity: Quantitative Cytokine (TNF-α and IL-6) Analysis

Lipopolysaccharide (LPS) stimulation was performed 2 days before the assay. PDLs were seeded in 200 μL of medium in 96-well microplates at 10^3^ cells/mL. The microplates were incubated at 37 °C in a controlled atmosphere containing 5% CO_2_ for 24 h. The day before the analysis, a *P. gingivalis* LPS solution (InvivoGen, San Diego, CA, USA) was added to the test wells at a 1 mg/mL concentration. The microplates were incubated for a further 24 h at 37 °C in a controlled atmosphere containing 5% CO_2_ [91].

On the day of the analysis, the media were removed from the wells and replaced with 200 μL of each extract [88,89]. The microplates were incubated for 24 h at 37 °C in a controlled atmosphere containing 5% CO_2_. One group of control cells (control group) was stimulated with LPS but not with an ICM extract. Two other groups of cells (culture control or positive control) were not stimulated with either LPS or the extracts.

TNF-α and IL-6 (proinflammatory markers) secretion by PDL cells was quantified in the supernatants and was analyzed using an enzyme-linked immunosorbent assay (ELISA) as previously described [93,94] (Supplementary Information S7).

### 4.5. Statistical Analysis

The values were tabulated using Microsoft Office Excel Mac OS 2011 (14.4.7 [141117] version). The significance level of the statistical analysis was set at *p* < 0.05. Multiple comparisons were performed with ANOVA and Tukey–Kramer post hoc tests using Microsoft Excel (MS Excel, Mac OS 201 16.74 version). All the experiments were repeated independently at least three times.

## 5. Conclusions

Within the limitations of this study, the proposed CH-based, CHX-loaded intracanal medications were effective against pathogens such as *Porphyromonas gingivalis* and *Enterococcus faecalis*, associated with combined endo-periodontal infections. Furthermore, the placement of these medications in the root canal resulted in a change in the physico-chemical conditions on the root surface related to the transradicular diffusion not only of ions (Ca^2+^, OH^−^) but also of the drug (CHX) contained in the ICM, mainly via the apex and slightly less via the dentinal tubules. The amounts of active compounds released through the dental root appeared non-toxic to periodontal cells in vitro and may also have an anti-inflammatory effect on stimulated periodontal ligament fibroblasts. Overall, these results provide a theoretical rationale to support the positive effects CH-based intracanal medications on the healing of endo-periodontal lesions.

## Figures and Tables

**Table 1:** (**A**) Antimicrobial activity of biofilm formation by *E. faecalis* with chlorhexidine-free calcium hydroxide paste (CH) and calcium hydroxide with 1% chlorhexidine (CH + CHX) every week for 4 weeks on Mueller–Hinton agar (MHA) and in brain heart infusion broth (*n* = 3). (**B**) Antimicrobial activity of the CH paste and the CHX paste on a mature *E. faecalis* biofilm after one week on MHA and in brain heart infusion broth (*n* = 3). (–) no bacterial growth, (+) bacterial growth.

A	Negative Control Samples		Positive Control Samples		Test Samples	
	CH	CH + CHX	CH	CH + CHX	CH	CH + CHX
Week 1	–	–	+	+	–	–
Week 2	–	–	+	+	–	–
Week 3	–	–	+	+	–	–
Week 4	–	–	+	+	–	–
**B**	**Negative Control Samples**		**Positive Control Samples**		**Test Samples**	
	**CH**	**CH + CHX**	**CH**	**CH + CHX**	**CH**	**CH + CHX**
Week 1	–	–	+	+	–	–

## Data Availability

The data presented in this study are available on request from the corresponding author.

## Supplementary Materials

The following supporting information can be downloaded at: https://www.mdpi.com/article/10.3390/antibiotics12091416/s1; S1: Minimal Inhibitory Concentrations and Minimal Bactericidal Concentrations; S2: Tooth Preparation; S3: Calcium Ion Release Analysis; S4: Chlorhexidine Release Analysis; S5: Alkaline Phosphatase Activity; S6: Mineralized Bone-Like Nodule Formation; S7: Anti-Inflammatory Activity: Quantitative Cytokine (TNF-α and IL-6) Analysis.

## References

[1] Herrera D., Retamal-Valdes B., Alonso B., Feres M. (2018). Acute periodontal lesions (periodontal abscesses and necrotizing periodontal diseases) and endo-periodontal lesions. J. Periodontol..

[2] Papapanou P.N., Sanz M., Buduneli N., Dietrich T., Feres M., Fine D.H., Flemmig T.F., Garcia R., Giannobile W.V., Graziani F. (2018). Periodontitis: Consensus report of workgroup 2 of the 2017 World Workshop on the Classification of Periodontal and Peri-Implant Diseases and Conditions. J. Clin. Periodontol..

[3] Altaf D.A., Jeelani D.M., Basher D.A. (2019). Assessment of prevalence of Endo-perio lesions among patients of known population: An observational study. Int. J. Appl. Dent. Sci..

[4] Prashaanthi N., Rajasekar A., Shantha Sundari K.K. (2021). Prevalence of endo perio lesion-an institutional study. Int. J. Dent. Oral Sci..

[5] Ruetters M., Gehrig H., Kronsteiner D., Schuessler D.L., Kim T.S. (2022). Prevalence of endo-perio lesions according to the 2017 World Workshop on the Classification of Periodontal and Peri-Implant Disease in a university hospital. Quintessence Int..

[6] Ahmed H.M.A. (2012). Different perspectives in understanding the pulp and periodontal intercommunications with a new proposed classification for endo-perio lesions. ENDO—Endod. Pract. Today.

[7] Rotstein I. (2017). Interaction between endodontics and periodontics. Periodontol. 2000.

[8] Siew K.L., Goh V., Goo C.L., Corbet E., Leung W.K. (2019). The Periodontal-Endodontic Relationship, What Do We Know?. Periodontol. Dent. Implantol..

[9] Karteva T., Manchorova-Veleva N. (2020). The Role of the Immune Response in Chronic Marginal and Apical Periodontitis. Folia Med..

[10] Gomes B.P.F.A., Montagner F., Berber V.B., Zaia A.A., Ferraz C.C.R., de Almeida J.F.A., Souza-Filho F.J. (2009). Antimicrobial action of intracanal medicaments on the external root surface. J. Dent..

[11] Singh P. (2011). Endo-perio dilemma: A brief review. Dent. Res. J..

[12] Solomon C., Chalfin H., Kellert M., Weseley P. (1995). The endodontic-periodontal lesion: A rational approach to treatment. J. Am. Dent. Assoc..

[13] Gambin D.J., Vitali F.C., De Carli J.P., Mazzon R.R., Gomes B.P.F.A., Duque T.M., Trentin M.S. (2021). Prevalence of red and orange microbial complexes in endodontic-periodontal lesions: A systematic review and meta-analysis. Clin. Oral Investig..

[14] Rovai E.d.S., Matos F.d.S., Kerbauy W.D., Cardoso F.G.d.R., Martinho F.C., Oliveira L.D., Valera M.C., Carvalho C.A.T. (2019). Microbial Profile and Endotoxin Levels in Primary Periodontal Lesions with Secondary Endodontic Involvement. Braz. Dent. J..

[15] Slots J. (2017). Periodontitis: Facts; fallacies and the future. Periodontol. 2000.

[16] Xia M., Qi Q. (2013). Bacterial analysis of combined periodontal-endodontic lesions by polymerase chain reaction-denaturing gradient gel electrophoresis. J. Oral Sci..

[17] Cucolo F., Bonvalente M.C., Barroso E.M., de Toledo B.E.C., Souza A.A., Zuza E.P. (2021). Endo-perio lesions prevalence in non-molar and molar teeth: A pilot study. Rev. Odontol. UNESP.

[18] Didilescu A.C., Rusu D., Anghel A., Nica L., Iliescu A., Greabu M., Bancescu G., Stratul S.I. (2012). Investigation of six selected bacterial species in endo-periodontal lesions. Int. Endod. J..

[19] Fan X., Xu X., Yu S., Liu P., Chen C., Pan Y., Lin L., Li C. (2020). Prognostic Factors of Grade 2-3 Endo-Periodontal Lesions Treated Nonsurgically in Patients with Periodontitis: A Retrospective Case-Control Study. Biomed. Res. Int..

[20] Tewari S., Sharma G., Tewari S., Mittal S., Bansal S. (2018). Effect of immediate periodontal surgical treatment on periodontal healing in combined endodontic-periodontal lesions with communication-A randomized clinical trial. J. Oral Biol. Craniofacial Res..

[21] Schmidt J.C., Walter C., Amato M., Weiger R. (2014). Treatment of periodontal-endodontic lesions—A systematic review. J. Clin. Periodontol..

[22] Mohammadi Z., Dummer P.M.H. (2011). Properties and applications of calcium hydroxide in endodontics and dental traumatology. Int. Endod. J..

[23] Siqueira J.F., Lopes H.P. (1999). Mechanisms of antimicrobial activity of calcium hydroxide: A critical review. Int. Endod. J..

[24] Leonardo M.R., Hernandez M.E.F.T., Silva L.A.B., Tanomaru-Filho M. (2006). Effect of a calcium hydroxide-based root canal dressing on periapical repair in dogs: A histological study. Oral Surg. Oral Med. Oral Pathol. Oral Radiol. Endod..

[25] De Rossi A., Silva L.A.B., Leonardo M.R., Rocha L.B., Rossi M.A. (2005). Effect of rotary or manual instrumentation; with or without a calcium hydroxide/1% chlorhexidine intracanal dressing; on the healing of experimentally induced chronic periapical lesions. Oral Surg. Oral Med. Oral Pathol. Oral Radiol. Endod..

[26] Gomes B.P.F.A., Vianna M.E., Zaia A.A., Almeida J.F.A., Souza-Filho F.J., Ferraz C.C.R. (2013). Chlorhexidine in endodontics. Braz. Dent. J..

[27] Mohammadi Z., Jafarzadeh H., Shalavi S., Sahebalam R., Kinoshita J.I. (2017). Additive and reducing Effects between Calcium Hydroxide and Current Irrigation Solutions. J. Contemp. Dent. Pract..

[28] Sy K., Agossa K., Maton M., Chijcheapaza-Flores H., Martel B., Siepmann F., Deveaux E., Blanchemain N., Neut C. (2021). How Adding Chlorhexidine or Metallic Nanoparticles Affects the Antimicrobial Performance of Calcium Hydroxide Paste as an Intracanal Medication: An In Vitro Study. Antibiotics.

[29] Raheja J., Tewari S., Tewari S., Duhan J. (2014). Evaluation of efficacy of chlorhexidine intracanal medicament on the periodontal healing of concomitant endodontic-periodontal lesions without communication: An interventional study. J. Periodontol..

[30] Shao W., Xiao F., Xu Z.X., Ren R.H., Wang Y., Wu Y.Q. (2018). Treatment of severe periodontic-endodontic combined lesions with minocycline hydrochloride ointment combined with mineral trioxide aggregate. Exp. Ther. Med..

[31] Duque T.M., Prado M., Herrera D.R., Gomes B.P.F.A. (2019). Periodontal and endodontic infectious/inflammatory profile in primary periodontal lesions with secondary endodontic involvement after a calcium hydroxide-based intracanal medication. Clin. Oral Investig..

[32] Carvalho C.N., Freire L.G., Carvalho A.P., Duarte M.A.H., Bauer J., Gavini G. (2016). Ions Release and pH of Calcium Hydroxide-; Chlorhexidine- and Bioactive Glass-Based Endodontic Medicaments. Braz. Dent. J..

[33] Chamberlain T.M., Kirkpatrick T.C., Rutledge R.E. (2009). pH changes in external root surface cavities after calcium hydroxide is placed at 1; 3 and 5 mm short of the radiographic apex. Dent. Traumatol..

[34] Esberard R.M., Carnes D.L., del Rio C.E. (1996). Changes in pH at the dentin surface in roots obturated with calcium hydroxide pastes. J. Endod..

[35] Miñana M., Carnes D.L., Walker W.A. (2001). pH Changes at the Surface of Root Dentin after Intracanal Dressing with Calcium Oxide and Calcium Hydroxide. J. Endod..

[36] Morfis A., Sylaras S.N., Georgopoulou M., Kernani M., Prountzos F. (1994). Study of the apices of human permanent teeth with the use of a scanning electron microscope. Oral Surg. Oral Med. Oral Pathol..

[37] Moreinos D., Front E., Lin S. (2021). Perio-endo interaction: A review. Oral Health Care.

[38] Barbin L.E., Estrela C., Guedes D.F.C., Spanó J.C.E., Sousa-Neto M.D., Pécora J.D. (2013). Detection of para-chloroaniline; reactive oxygen species; and 1-chloro-4-nitrobenzene in high concentrations of chlorhexidine and in a mixture of chlorhexidine and calcium hydroxide. J. Endod..

[39] Below H., Assadian O., Baguhl R., Hildebrandt U., Jäger B., Meissner K., Leaper D.J., Kramer A. (2017). Measurements of chlorhexidine; p-chloroaniline; and p-chloronitrobenzene in saliva after mouth wash before and after operation with 0.2% chlorhexidine digluconate in maxillofacial surgery: A randomised controlled trial. Br. J. Oral Maxillofac. Surg..

[40] Câmara De Bem S.H., Estrela C., Guedes D.F.C., Sousa-Neto M.D., Pécora J.D. (2014). Determination of chemical components derived from 2% chlorhexidine gel degradation using gas chromatography-mass spectrometry. Acta Odontol. Scand..

[41] Khatib M.S., Ameer B., Ajit Mannur N., Ramalingaiahsetty A.M., Peerzade S.M., Bambawale A. (2020). Decoding the Perplexing Mystery of Para-Chloroaniline Formation: A Systematic Review. J. Int. Soc. Prev. Community Dent..

[42] Selvamani M., Madhushankari G.S., Basandi S.P., Donoghue M., Nayak V., Diwakar G. (2013). Effect of Vitality on Translucent Dentine—A Study. J. Int. Oral Health.

[43] Weber D.F. (1974). Human dentine sclerosis: A microradiographic survey. Arch. Oral Biol..

[44] Jacinto R.C., Linhares-Farina G., Sposito O.d.S., Zanchi C.H., Cenci M.S. (2015). Influence of 2% chlorhexidine on pH; calcium release and setting time of a resinous MTA-based root-end filling material. Braz. Oral Res..

[45] Krüger H.C., Francio J., Silva A.S., da Oliveira G.S.N., de Brancher J.A., Dantas L.R., Oliveira R.C., Tuon F.F., Carneiro E. (2021). Antimicrobial action; cytotoxicity; calcium ion release; and pH variation of a calcium hydroxide-based paste associated with Myracrodruon urundeuva Allemão extract. Aust. Endod. J. J. Aust. Soc. Endodontology Inc..

[46] Misra P., Bains R., Loomba K., Singh A., Sharma V.P., Murthy R.C., Kumar R. (2017). Measurement of pH and calcium ions release from different calcium hydroxide pastes at different intervals of time: Atomic spectrophotometric analysis. J. Oral Biol. Craniofac. Res..

[47] Ximenes M., Cardoso M. (2012). Assessment of diffusion of hydroxyl and calcium ions of root canal filling materials in primary teeth. Pediatr. Dent..

[48] Guerreiro-Tanomaru J.M., Chula D.G., de Pontes Lima R.K., Berbert F.L.V.C., Tanomaru-Filho M. (2012). Release and diffusion of hydroxyl ion from calcium hydroxide-based medicaments. Dent. Traumatol..

[49] Pacios M.G., de la Casa M.L., de Bulacio M.l., López M.E. (2004). Influence of different vehicles on the pH of calcium hydroxide pastes. J. Oral Sci..

[50] Tanomaru J.M.G., Leonardo M.R., Tanomaru Filho M., Bonetti Filho I., Silva L.a.B. (2003). Effect of different irrigation solutions and calcium hydroxide on bacterial LPS. Int. Endod. J..

[51] Duarte M.A.H., Midena R.Z., Zeferino M.A., Vivan R.R., Weckwerth P.H., Dos Santos F., Guerreiro-Tanomaru J.M., Tanomaru-Filho M. (2009). Evaluation of pH and calcium ion release of calcium hydroxide pastes containing different substances. J. Endod..

[52] Signoretti F.G.C., Gomes B.P.F., Montagner F., Barrichello Tosello F., Jacinto R.C. (2011). Influence of 2% chlorhexidine gel on calcium hydroxide ionic dissociation and its ability of reducing endotoxin. Oral Surg. Oral Med. Oral Pathol. Oral Radiol. Endod..

[53] Lima T.F.R., Ascendino J.F., Cavalcante I.d.O.D., Assunção F.L.C., Salazar-Silva J.R., da Silva E.J.N.L., Lemos S.G., Soares A.J. (2019). Influence of chlorhexidine and zinc oxide in calcium hydroxide pastes on pH changes in external root surface. Braz. Oral Res..

[54] Delgado R.J.R., Gasparoto T.H., Sipert C.R., Pinheiro C.R., Moraes I.G., Garcia R.B., Bramante C.M., Campanelli A.P., Bernardineli N. (2010). Antimicrobial effects of calcium hydroxide and chlorhexidine on *Enterococcus faecalis*. J. Endod..

[55] Balto H., Bukhary S., Al-Omran O., BaHammam A., Al-Mutairi B. (2020). Combined Effect of a Mixture of Silver Nanoparticles and Calcium Hydroxide against *Enterococcus faecalis* Biofilm. J. Endod..

[56] Blanscet M.L., Tordik P.A., Goodell G.G. (2008). An agar diffusion comparison of the antimicrobial effect of calcium hydroxide at five different concentrations with three different vehicles. J. Endod..

[57] da Silva R.A.B., Leonardo M.R., da Silva L.A.B., de Castro L.M.S., Rosa A.L., de Oliveira P.T. (2008). Effects of the association between a calcium hydroxide paste and 0.4% chlorhexidine on the development of the osteogenic phenotype in vitro. J. Endod..

[58] Voel D., Voel J.G., Pratt C.W. (2016). Fundamentals of Biochemistry.

[59] De-Deus G., Canabarro A., Alves G., Linhares A., Senne M.I., Granjeiro J.M. (2009). Optimal cytocompatibility of a bioceramic nanoparticulate cement in primary human mesenchymal cells. J. Endod..

[60] Garg A., Mala K., Kamath P.M. (2021). Biofilm models in endodontics—A narrative review. J. Conserv. Dent..

[61] Tülü G., Kaya B.Ü., Çetin E.S., Köle M. (2021). Antibacterial effect of silver nanoparticles mixed with calcium hydroxide or chlorhexidine on multispecies biofilms. Odontology.

[62] Kim D., Kim E. (2014). Antimicrobial effect of calcium hydroxide as an intracanal medicament in root canal treatment: A literature review—Part I. In vitro studies. Restor. Dent. Endod..

[63] Swimberghe R.C.D., Coenye T., De Moor R.J.G., Meire M.A. (2019). Biofilm model systems for root canal disinfection: A literature review. Int. Endod. J..

[64] Afkhami F., Ahmadi P., Chiniforush N., Sooratgar A. (2021). Effect of different activations of silver nanoparticle irrigants on the elimination of *Enterococcus faecalis*. Clin. Oral Investig..

[65] Choi M.J., Kim M.A., Choi Y., Neelakantan P., Yu M.K., Min K.S. (2021). A novel three-dimensionally printed model to assess biofilm removal by ultrasonically activated irrigation. Int. Endod. J..

[66] Hoedke D., Kaulika N., Dommisch H., Schlafer S., Shemesh H., Bitter K. (2021). Reduction of dual-species biofilm after sonic- or ultrasonic-activated irrigation protocols: A laboratory study. Int. Endod. J..

[67] Ma J., Tong Z., Ling J., Liu H., Wei X. (2015). The effects of sodium hypochlorite and chlorhexidine irrigants on the antibacterial activities of alkaline media against *Enterococcus faecalis*. Arch. Oral Biol..

[68] Li Y., Wang Y., Chen X., Jiang W., Jiang X., Zeng Y., Li X., Feng Z., Luo J., Zhang L. (2020). Antimicrobial peptide GH12 as root canal irrigant inhibits biofilm and virulence of *Enterococcus faecalis*. Int. Endod. J..

[69] Qaeed M.A., Hendi A., Obaid A.S., Thahe A.A., Osman A.M., Ismail A., Mindil A., Eid A.A., Aqlan F., Osman N.M.A. (2023). The effect of different aqueous solutions ratios of *Ocimum basilicum* utilized in AgNPs synthesis on the inhibition of bacterial growth. Sci. Rep..

[70] Bekhouche M., Bolon M., Charriaud F., Lamrayah M., Da Costa D., Primard C., Costantini A., Pasdeloup M., Gobert S., Mallein-Gerin F. (2020). Development of an antibacterial nanocomposite hydrogel for human dental pulp engineering. J. Mater. Chem. B.

[71] Pintor A.V.B., Queiroz L.D., Sancas M.C., Brochado A.C.B., Spoladore J., Fonseca-Gonçalves A., Fidalgo T.K.S., Freitas-Fernandes L.B., Valente A.P., de Souza I.P.R. (2021). Cytocompatibility of filling pastes by primary teeth root simulating model. Odontology.

[72] Kranz S., Guellmar A., Braeutigam F., Tonndorf-Martini S., Heyder M., Reise M., Sigusch B. (2021). Antibacterial Effect of Endodontic Disinfections on *Enterococcus faecalis* in Dental Root Canals-An In-Vitro Model Study. Materials.

[73] Mounir M.M.F., Rashed F.M., Bukhary S.M. (2022). Amelogenin as a regenerative endodontic molecule for immature teeth with apical periodontitis. An experimental study. J. Oral Biol. Craniofac. Res..

[74] Li M., Yang Y., Lin C., Zhang Q., Gong L., Wang Y., Zhang X. (2021). Antibacterial Properties of Small-Size Peptide Derived from Penetratin against Oral *Streptococci*. Materials.

[75] Abedini A., Roumy V., Mahieux S., Gohari A., Farimani M.M., Rivière C., Samaillie J., Sahpaz S., Bailleul F., Neut C. (2014). Antimicrobial activity of selected Iranian medicinal plants against a broad spectrum of pathogenic and drug multiresistant micro-organisms. Lett. Appl. Microbiol..

[76] Shetty S., Sekar P., Shetty R.M., Abou Neel E.A. (2023). Antibacterial and Antibiofilm Efficacy of Copper-Doped Phosphate Glass on Pathogenic Bacteria. Molecules.

[77] Bago Jurič I., Plečko V., Anić I., Pleško S., Jakovljević S., Rocca J.P., Medioni E. (2016). Antimicrobial efficacy of photodynamic therapy; Nd:YAG laser and QMiX solution against *Enterococcus faecalis* biofilm. Photodiagnosis Photodyn. Ther..

[78] Raoof M., Khaleghi M., Siasar N., Mohannadalizadeh S., Haghani J., Amanpour S. (2019). Antimicrobial Activity of Methanolic Extracts of *Myrtus communis* L. and *Eucalyptus Galbie* and their Combination with Calcium Hydroxide Powder against *Enterococcus faecalis*. J. Dent. Shiraz Iran..

[79] Song Z.M., Zhang J.L., Zhou K., Yue L.M., Zhang Y., Wang C.Y., Wang K.L., Xu Y. (2021). Anthraquinones as Potential Antibiofilm Agents Against Methicillin-Resistant *Staphylococcus aureus*. Front. Microbiol..

[80] Eftekhar B., Moghimipour E., Eini E., Jafarzadeh M., Behrooz N. (2014). Evaluation of hydroxyl ion diffusion in dentin and injectable forms and a simple powder-water calcium hydroxide paste: An in vitro study. Jundishapur J. Nat. Pharm. Prod..

[81] Forghani M., Mashhoor H., Rouhani A., Jafarzadeh H. (2014). Comparison of pH changes induced by calcium enriched mixture and those of calcium hydroxide in simulated root resorption defects. J. Endod..

[82] Shetty S., Manjunath M.K., Tejaswi S. (2014). An In-vitro Evaluation of the pH Change Through Root Dentin Using Different Calcium Hydroxide Preparations as an Intracanal Medicament. J. Clin. Diagn. Res. JCDR.

[83] Yazdanpanahi N., Behzadi A., Zare Jahromi M. (2021). Long-term pH Alterations in the Periradicular Area Following the Application of Calcium Hydroxide and MTA. J. Dent. Shiraz Iran..

[84] Lizzi F., Goutaudier C., Attik N., Jackson P., Campbell I., Mokbel I., Grosgogeat B., Villat C. (2020). Ion release characterization in phase separated borosilicate glass powders. J. Non-Cryst. Solids.

[85] Kudo K., Ikeda N., Kiyoshima A., Hino Y., Nishida N., Inoue N. (2002). Toxicological analysis of chlorhexidine in human serum using HPLC on a polymer-coated ODS column. J. Anal. Toxicol..

[86] Xue Y., Tang M., Hieda Y., Fujihara J., Takayama K., Takatsuka H., Takeshita H. (2009). High-performance liquid chromatographic determination of chlorhexidine in whole blood by solid-phase extraction and kinetics following an intravenous infusion in rats. J. Anal. Toxicol..

[87] Cardoso M.A., Fávero M.L.D., Gasparetto J.C., Hess B.S., Stremel D.P., Pontarolo R. (2011). Development and Validation of an Rp-Hplc Method for the Determination of Chlorhexidine and P-Chloroaniline in Various Pharmaceutical Formulations. J. Liq. Chromatogr. Relat. Technol..

[88] Peng W., Huan Z., Pei G., Li J., Cao Y., Jiang L., Zhu Y. (2022). Silicate bioceramics elicit proliferation and odonto-genic differentiation of human dental pulp cells. Dent. Mater. J..

[89] Mukhtar-Fayyad D. (2011). Cytocompatibility of new bioceramic-based materials on human fibroblast cells (MRC-5). Oral Surg. Oral Med. Oral Pathol. Oral Radiol. Endod..

[90] Zhou Y.H., Xie Q. (2021). Total glycosides from Eucommia ulmoides seed promoted osteogenic differentiation of adipose-derived mesenchymal stem cells and bone formation in ovariectomized rats through regulating Notch signaling pathway. J. Orthop. Surg..

[91] Guerreiro J.C.M., Ochoa-Rodrígez V.M., Rodrigues E.M., Chavez-Andrade G.M., Tanomaru-Filho M., Guerreiro-Tanomaru J.M., Faria G. (2021). Antibacterial activity; cytocompatibility and effect of Bio-C Temp bioceramic intracanal medicament on osteoblast biology. Int. Endod. J..

[92] Narita H., Itoh S., Imazato S., Yoshitake F., Ebisu S. (2010). An explanation of the mineralization mechanism in osteoblasts induced by calcium hydroxide. Acta Biomater..

[93] Morand D.N., Huck O., Keller L., Jessel N., Tenenbaum H., Davideau J.L. (2015). Active Nanofibrous Membrane Effects on Gingival Cell Inflammatory Response. Materials.

[94] Jeanneau C., Giraud T., Laurent P., About I. (2019). BioRoot RCS Extracts Modulate the Early Mechanisms of Periodontal Inflammation and Regeneration. J. Endod..

