# Supplementary material for: Therapeutic Potential of Chlorhexidine-Loaded Calcium Hydroxide-Based Intracanal Medications in Endo-Periodontal Lesions: An Ex Vivo and In Vitro Study
Source: Antibiotics (Basel). 2023 Sep 7;12(9):1416. doi: 10.3390/antibiotics12091416 (PMC10525524; doi:10.3390/antibiotics12091416)
Supplement: Supplementary file 1 [file antibiotics-12-01416-s001.zip › antibiotics-2514312-supplementary.pdf]

Supplementary Information

S1. Minimal Inhibitory Concentrations and Minimal Bactericidal Concentrations

The MIC and MBC of *P. gingivalis* were determined in triplicate, with antimicrobial concentrations ranging from 1 to 512 mg/L. First, 1.6 mL of Mueller-Hinton Broth (MH) was added to each well. The CHX solution (5,120 mg/L) was serially diluted two-fold in distilled water and 0.2 mL was added to the corresponding well. The microplates were then inoculated with 0.2 mL/well of fresh bacterial culture at a concentration of 10^6 bacteria/mL and were incubated at 37°C under strict anaerobic conditions. The MIC was defined as the lowest concentration that inhibited visible growth (turbidity) after 48 h. Bacterial suspensions (0.1 mL) at a determined MIC as well as the two following concentrations were seeded on Mueller Hinton Agar plates to determine the MBCs. The MBCs were the lowest concentrations at which no colony formation occurred.

S2. Tooth Preparation

Teeth with decay, cracks, attrition, or abrasion were excluded. The teeth were placed in 0.5% chloramine-T (Sigma-Aldrich) for 7 days and were then stored in normal saline solution (Alfa Aesar®, Kandel, Germany) at 4°C until used. Periapical radiographs (in the mesiodistal and buccolingual directions) were taken to confirm the presence of a single canal [32]. The teeth were decoronated 11 to 12 mm from the apex to create equal length roots using a sanding disc (Buehler MetaServ® 250). The working length was confirmed using a #15 K-file (Dentsply Maillefer, Ballaigues, Switzerland). This procedure was followed by canal instrumentation using a ProTaper rotary system (Dentsply Maillefer) in accordance with the manufacturer's instructions. The canals were irrigated between each instrumentation with an apical master file F3 (size 3, taper 0.05) using 2.5 mL of 2.5% sodium hypochlorite [78,79]. Five mL of 17% EDTA solution was prepared from EDTA powder by diluting 17.36 mg in 100 mL of distilled water. The solution was used to remove the smear layer by irrigating the canal for 1 min [32,79] followed by distilled water and 2.5 mL of 2.5% NaOCl [53]. The canals were dried with paper tips (Coltène/Whaledent, Le Mans, France) and the external surfaces with filter paper [32].

S3. Calcium Ion Release Analysis

Two mL of the release medium from each sample was filtered upstream through a 0.45-µm nylon filter (VWR, International, Radnor, PA, USA). Calibration curves were obtained by preparing standard 2, 5, 10, and 20 ppm Ca^{2+} solutions. The measurements were taken on days 0, 7, and 28 [32].

S4. Chlorhexidine Release Analysis

A 20-µL sample was injected onto a C18 RP column (Gemini-NX, 5 µm C18 110 Å, 250 mm x~4.6 mm; Phenomenex, Le Pecq, France). The mobile phase was an aqueous solution of 0.05% trifluoroacetic acid, 0.05% heptafluorobutyric acid, 0.1% triethylamine/acetonitrile 60/40 (*v*/*v*). The flow rate was 1 mL/min. The column was kept at room temperature, and the detection wavelength was set at λ = 254 nm.

S5. Alkaline Phosphatase Activity

Fifty µL of thymolphthalein monophosphate was mixed with 0.5 mL of 0.3 mol/L diethanolamine buffer (pH 10.1) and was incubated for 2 min at 37°C. The solution was then added to 50 µL of the lysates in each well. The microplates were incubated for 10 min at 37°C. For color staining, 200 µL of 0.09 mol/L anhydrous sodium carbonate (Na_2CO_3) and 0.25 mol/L sodium hydroxide (NaOH) were added. The ALP in the samples converts p-nitrophenyl phosphate (pNPP) to an equal amount of colored p-nitrophenol (pNP). After 60 min, the absorbance was measured at 405 nm, and ALP activity was calculated from a standard curve using thymolphthalein to give a range of 0.012-0.4 µmoL of thymolphthalein/h/mL.

Lastly, ALP activity was calculated using the following equation:

$$\text{ALP activity} = \frac{[\text{pNP}]}{\Delta T \times V} \times D$$

where [pNP] is the amount of pNP in μmoL calculated from the standard curve, ΔT is the reaction time (min), V is the initial volume added to each well, and D is the dilution factor.

S6. Mineralized Bone-Like Nodule Formation

ARS targets calcium deposits in the extracellular matrix. The cells were fixed with 3.7% paraformaldehyde for 30 min at room temperature, rinsed with PBS, washed with distilled water, and incubated with 100 μL of 40 mM ARS (2%, pH 4.1; Sigma-Aldrich) for 45 min at room temperature. A qualitative test was performed by observing the mineralization nodules under an optical microscope. Inorganic precipitates were semi-quantified by dissolving the mineralized matrix nodules generated by the cells with 200 μL of a 10% cetylpyridinium chloride solution (Sigma-Aldrich) for 30 min at room temperature with stirring. Optical density values representing the relative amounts of mineralization nodules were determined at 560 nm.

S7. Anti-Inflammatory Activity: Quantitative Cytokine (TNF-α and IL-6) Analysis

The cells were centrifuged for at 4°C for 10 min at 13,000g. The mediator release assay was carried out according to the manufacturer's instructions using human (for PDL and MG63) or mouse (for OCM) TNF-α uncoated ELISA kits (Invitrogen, Thermo Fisher Scientific) for TNF-α and human (for PDLs and MG63) or mouse (for OCM) IL-6 (Elabscience®, Houston, TX, USA). The experiment was performed in triplicate, and the data were compared to a standard curve. Optical density values were obtained using a microplate reader (Tecan®, Lyon, France), at 450 nm, with a reference at 570 nm for the TNF-α kits. The local degradation of pro-inflammatory mediators in a basic environment may be one of the anti-inflammatory mechanisms by which CH contributes to the healing of periapical inflammatory lesions.
